# Essential Thrombocythemia and Acquired von Willebrand Syndrome: The Shadowlands between Thrombosis and Bleeding

**DOI:** 10.3390/cancers12071746

**Published:** 2020-06-30

**Authors:** Hassan Awada, Maria Teresa Voso, Paola Guglielmelli, Carmelo Gurnari

**Affiliations:** 1 Department of Translational Hematology and Oncology Research, Taussig Cancer Institute, Cleveland Clinic, Cleveland, OH 44106, USA; carmelogurnari31@gmail.com; 2 Department of Biomedicine and Prevention, University of Rome Tor Vergata, 00133 Rome, Italy; Voso@med.uniroma2.it; 3 Fondazione Santa Lucia, Laboratorio di Neuro-Oncoematologia, 00143 Roma, Italy; 4 CRIMM-Centro Ricerca e Innovazione delle Malattie Mieloproliferative, Department of Experimental and Clinical Medicine, Azienda ospedaliera-Universitaria Careggi, University of Florence, 50139 Florence, Italy; paola.guglielmelli@unifi.it

**Keywords:** essential thrombocythemia, cytoreductive therapy, excessive thrombocytosis, acquired von Willebrand syndrome

## Abstract

Over the past decade, new insights have emerged on the pathophysiology of essential thrombocythemia (ET), its clinical management, and associated thrombohemostatic disturbances. Here, we review the latest diagnostic and risk stratification modalities of ET and its therapeutics. Moreover, we discuss the clinical evidence-based benefits, deriving from major clinical trials, of using cytoreductive therapy and antiplatelet agents to lower the risk of fatal vascular events. Also, we focus on the condition of extreme thrombocytosis (>1000 × 10^9^/L) and bleeding risk, the development and pathogenesis of acquired von Willebrand syndrome, and the clinical approach to this paradoxical scenario in ET.

## 1. Introduction

Essential thrombocythemia (ET) is a clonal hematopoietic stem cell disorder that belongs to Philadelphia chromosome-negative myeloproliferative neoplasm (MPN) category [1,2]. ET is phenotypically expressed by persistent nonreactive thrombocytosis and increased risk of vascular events, resulting from the clonal proliferation of atypical megakaryocytes in the bone marrow. The prevalence of ET is estimated to be 38–57 per 100,000 population in the United States with a median age of 58 years on presentation and a female sex preponderance [3]. ET shares with other MPNs the classical driver mutations affecting genes like Janus kinase (*JAK2* V617F; 50–60%), calreticulin (*CALR*; 25–35%), or myeloproliferative leukemia (*MPL*; 5–10%) [4,5,6,7]. Given the great deal of genotypic and phenotypic overlap with other MPN subtypes, the diagnosis of ET can be challenging in some situations. For instance, *JAK2* V617F mutations and isolated thrombocytosis can be also seen in myelofibrosis (MF) and polycythemia vera (PV). [8,9] Additionally, it is of paramount importance to distinguish ET from prefibrotic myelofibrosis (PFMF), as the clinical picture of PFMF may resemble ET but its prognosis and management is more related to that of MF [10]. Hence, a stringent standard for ET diagnoses has been implemented and sequentially updated by the World Health Organization (WHO) with the latest 2016 revised version requiring the satisfaction of four major criteria, or three major criteria and one minor criterion to confirm the diagnosis [2] (Table 1). In fact, the presence of driver mutations influences disease evolution and has diagnostic and prognostic significance in ET [11,12,13]. Nevertheless, 10–20% of the patients are wild type for the aforementioned somatic hits (denoted hereafter as “triple-negative”) and express no driver mutations [14]. Interestingly, two studies have shown that about 8–10% of triple-negative patients carry activating mutations of *JAK2* or *MPL* outside of the classical loci and these non-canonical mutations may be either acquired (somatic) or inherited (germline) suggesting that they are more likely benign disorders of platelet production rather than MPNs [15,16].

Indeed, ET is a chronic disorder with a 15-year survival rate of 80% and a natural history that is characterized by the potential for progression to MF as well as secondary acute myeloid leukemia (sAML) [17,18]. Furthermore, morbidity and mortality are more associated with vascular risks (thrombosis and hemorrhage), hence, the primary goal of current prognostication models and available therapeutics is mainly to identify and appropriately manage patients at higher risk of such complications. Finally, recent insights deriving from next-generation sequencing (NGS) revealed that 53% of an ET cohort (*n* = 183) had sequence variants/mutations other than the classical driver mutations, with *TET2* and *ASXL1* being the most frequently mutated genes [19]. Hits affecting other myeloid genes like *SH2B3*, *IDH2*, *SF3B1*, *U2AF1*, *EZH2,* and *TP53* were found to have an adverse impact on the overall, leukemia-free, MF-free survival as well as an increased vascular risk in the studied ET population [19]. These observations raise the question of whether we need to implement the use of targeted NGS in routine management, surveillance, and design of therapeutic trajectory of patients with ET.

## 2. Clinical and Therapeutic Pitfalls

While the heterogeneous molecular profile of ET tend to drive the disease evolution, the phenotype, including the thrombo-hemorrhagic tendency and systemic symptom burden (e.g., fatigue, pruritus, microvascular symptoms, splenomegaly) [20], remains the major target of cytoreductive and antiplatelet therapies [21]. Hence, the goal is to relieve symptoms and decrease fatal vascular complications in a preventive fashion *via* lowering the persistent platelet elevation, ideally to less than 400 × 10^9^/L. 

ET comprises a wide spectrum of clinical complications, including thrombosis of major vessels, deep venous thrombosis or pulmonary embolism as well as other unusual sites [22,23,24]. The latter clinical scenario is more frequent among *JAK2* V617F carriers and may represent the first sign of disease onset; e.g., development of thrombosis in the splanchnic vessels (Budd-Chiari syndrome) or cerebral venous sinus [25]. ET patients also suffer from microvascular occlusions involving small vessels, which can cause ocular migraine (amaurosis fugax), transient ischemic attack, or erythromelalgia [26,27]. On the other hand, minor bleeding or major hemorrhagic complications can paradoxically happen, especially with extreme thrombocytosis [28,29,30]. While this may be true, vascular complications tend to controversially correlate with the extent and degree of thrombocytosis. For instance, a platelet count (PC) > 1000 × 10^9^/L can induce an acquired von Willebrand syndrome (AVWS) [31], caused by the proteolytic reduction of von Willebrand factor (VWF) multimers due to the passive adsorption to the platelet membrane. In contrast, lower PC (<1000 × 10^9^/L) has been associated with arterial and venous thrombosis (ischemic stroke, deep venous thrombosis, pulmonary embolism, etc.), with an increased risk observed when *JAK2* mutation is present [6,32]. All these observations illustrate the complexity of the disease and its intricate nature, as well as the controversy related to the therapeutic interventions in the clinical setting. In addition, as previously mentioned, patients with ET are at higher risk of fatal vascular events and the main therapeutic treatment, which is cytoreduction, only prevents vascular complications without reducing the risk of leukemic transformation or fibrotic progression [33].

## 3. Risk Factors and Stratification Models

### 3.1. The Evolution of Prognostication Systems

The higher thrombotic or bleeding risk in ET is not always well-defined because of commonalities and shadowlands in the clinical presentation of such situations, taking into account the possible simultaneous evolution of a case of extreme thrombocytosis. So far, patients with ET have been stratified by various models for vascular complications’ risk (mainly thrombotic) using factors like age (<40, 40–59 and 60 years), history of thrombosis, and *JAK2*/*MPL*/*CALR* mutation status and assessed for cardiovascular risk factors including hypertension, diabetes, and others. 

Historically, the European LeukemiaNet (ELN) categorized ET patients into high and low-risk groups according to a two-tiered model that takes into consideration the absence or presence of either age >60 years or history of thrombosis. Patients not meeting any of these two factors, fall in the low-risk category. In 2012, Barbui et al. proposed a 3-tiered score (with the addition of an intermediate-risk group) called the “International Prognostic Score of Thrombosis for Essential Thrombocythemia” (IPSET-thrombosis model), which accounted for the presence of cardiovascular risk factors and *JAK2* V617F mutation [34]. A more recent risk stratification modality is the new version of IPSET model that revised the individualized and combined contribution of cardiovascular risk factors and *JAK2*/*MPL* mutations and divided ET patients into 4-tiered risk categories, a) very-low-risk patients, who have no adverse features (age <61 years, no prior history of thrombosis or major hemorrhage, and absent *JAK2* V617F/*MPL* mutations), b) low-risk patients, who harbor *JAK2* V617F/*MPL* mutations but no adverse features, c) intermediate-risk patients, who are older (age >60 or older), but have no prior history of thrombosis or major hemorrhage and lack *JAK2* V617F/*MPL* mutations, and d) high-risk patients, who have a thrombosis history, or are older and harbor *JAK2* V617F/*MPL* mutations [35,36,37] (Table 2). As concluded from the serial revisions of the prognostication models, thrombosis risk may vary according to the mutational status. For instance, the integration of *MPL* mutations in the revised IPSET model relied on the association of this mutation with older age (a main risk factor for ET prognosis) [38]. Although *MPL*-mutated patients share a similar clinical picture with *CALR*-mutants, the latter group has a lower incidence of thrombosis, especially when compared to *JAK2* V617F mutated counterpart (10.5 *vs*. 25.1%, respectively; *p* = 0.01) [39]. As a matter of fact, also *JAK2* V617F mutant allele burden has been related to both arterial (values >25%; *p* = 0.055) and venous (values > 90%; *p* = 0.036) thrombosis [40]. In the meantime, Passamonti et al. proposed a prognostic model (IPSET-survival) based on age (≥60 years), leukocyte count (WBC; ≥11 × 10^9^/L), and history of thrombosis which enabled survival prediction at the time of diagnosis [41]. The IPSET-survival model consented to stratify ET patients into categories of significantly different survivals with median values ranging from 18.6 years in the low-risk category, to 7.1 years in the high-risk category.

Little is known about the other non-driver mutations’ impact on the vascular risk assessment; however, the enrichment in *TET2* mutations in ET has been identified to be associated with a higher incidence of thrombotic risk [19]. After the new insights on the molecular characterization of MF, which led to the development of newer risk models that included genetic biomarkers such as MIPSS70 (Mutation-enhanced international prognostic systems) [42], Tefferi and colleagues developed a similar approach for ET and PV called the “MIPSS-ET/PV” [43]. After analyzing data from two different cohorts in this recent study, multivariable analysis confirmed an independent survival effect associated with the presence of adverse mutations (*SRSF2*, *SF3B1*, *U2AF1*, and *TP53*), age >60 years, male sex and a leukocyte count ≥11 × 10^9^/L in ET patients. This led to a 3-tiered model that has been shown to be superior to the conventional IPSET-survival score in predicting survival rates of patients with ET. The main difference from the previous models is that thrombosis history is no longer considered a risk factor (Table 2).

### 3.2. Mutational Profile

As discussed, due to the chronic manifestation of this disease, the main goal of ET management remains the prevention of fatal vascular complications which have been reported to be the leading cause of death [31]. Up to 24% of ET patients develop a vascular event before (13%) or after (11%) diagnosis with a lower rate in *CALR*-mutants as compared to *JAK2* V617F/*MPL* mutants and triple-negative cases [39,44]. Apart from having different molecular lesions and survival outcomes, *JAK2* V617F-mutated ET patients had a higher hemoglobin level and WBC (the so-called “PV-like phenotype”), and a lower PC while a substantial fraction of *CALR*-mutants had a PC >1000 × 10^9^/L [39]. Despite this association, *CALR*-mutated patients had a lower risk of thrombosis, pointing out that the PC per se is not the only determinant of vascular complications [39]. Platelets from patients with *CALR* mutations were significantly less activated following adenosine diphosphate (ADP) stimulation compared to that of a control group or *JAK2* mutants (*p* < 0.001) [45]. Moreover, among the two predominant variants of *CALR* mutations (type 1 and 2), patients harboring type 2 *CALR* mutations seemed to have a more indolent disease course [46]. 

### 3.3. Leukocytosis

As observed in PV, many studies demonstrated a correlation between leukocytosis and vascular events in ET [28]. For instance, it has been observed that a near to linear correlation between thrombosis and WBC existed in ET patients [10]. A recent meta-analysis highlighted the dual effect of leukocytosis on both thrombosis and bleeding events [47]. Moreover, this negative impact seemed to be independent of the PC per se as observed in patients treated with anagrelide with optimized PC < 575 × 10^9^/L but WBC > 9.66 × 10^9^/L [48]. This observation emphasizes the role of WBC beyond PC per se on triggering vascular complications, as highlighted and taken into consideration by risk assessment models. Finally, we might assume that the use of anagrelide, not providing a normalization of WBC, should be reserved for lower-risk patients without evidence of leukocytosis.

### 3.4. Inherited Thrombophilia

As aforementioned, about 25% of patients with ET experience an episode of thrombosis during the disease course. In this context, it is also important to identify possible carriers of other inherited thrombophilic risk-factors, including antithrombin and protein C functional activities, free protein S antigen, fasting homocysteine, Factor V Leiden, PT G20210A, and antiphospholipid antibodies (lupus anticoagulant, anticardiolipin antibodies, and anti-β2glycoprotein I). For example, De Stefano et al [49] reported that younger patients with ET are at higher risk of thrombotic events in the case of the concomitant presence of both *JAK2* V617F mutation and inherited thrombophilia (5-fold increase as compared to non-carriers of either alteration). Particularly, Factor V Leiden seemed to be associated with an increased thrombotic risk in patients with ET [50]. Nevertheless, literature reports have been controversial and failed to demonstrate a higher prevalence of thrombophilic conditions in patients with ET. 

## 4. Cytoreductive Therapies

Risk stratification is an early step that follows diagnosis and is critically important to guide clinicians towards appropriate therapeutic interventions. Cytoreductive therapies (e.g., hydroxyurea (HU), anagrelide, pegylated interferon α (P-IFNα), pipobroman, busulphan, and radioactive phosphorus) remain the backbone of treatments available for ET patients of the high-risk group. The specific drug of choice is basically selected according to the individual risk, defined as already discussed, by age, past medical history, type of driver mutation and, last but not least, the patient’s preferences in the context of a patient-physician therapeutic alliance. A detailed description of all the cytoreductive agents and their main conducted clinical trials in ET are reviewed below.

### 4.1. Hydroxyurea

The PT-1 randomized controlled trial (RCT) led to the wide use of HU as frontline therapy [51]. The study enrolled 809 high-risk patients with ET and showed that HU is superior to anagrelide in reducing arterial thrombosis, serious hemorrhage, and myelofibrotic progression. When combined with aspirin (ASA), HU decreased the rate of thrombotic events by 20.4% compared with single-agent ASA in a study of 114 patients [52]. However, the phase III ANAHYDRET trial concluded that anagrelide, as a selective platelet-lowering agent, was not inferior to HU in preventing thrombotic complications when ET patients were diagnosed according to the 2008 WHO criteria [53]. 

### 4.2. Interferons

An alternative frontline therapy is the recombinant interferon α [e.g., interferon α-2b, pegylated interferon α-2a (P-IFNα-2a), and pegylated interferon α-2b (P-IFNα-2b)] which has been traditionally used in ET and had successful hematologic and molecular responses [54,55]. Although the hematologic response was not different, *JAK2* V617F-positive patients were more likely to achieve higher molecular response with P-IFNα, as compared to *CALR*-mutant individuals [55] and according to this evidence, a recent study suggested that higher doses are needed in the latter population [56]. Long-term efficacy and safety data of P-IFNα-2a treatment were reported in a single-center, open-label, phase 2 trial after a median follow up of 83 months [57]. The study showed that P-IFNα-2a can produce durable hematological and molecular responses in ET patients. Moreover, the Myeloproliferative Neoplasms Research Consortium (MPN-RC) 112 randomized trial that compared P-IFNα-2a vs. HU for the treatment of high-risk PV and ET, revealed that complete response rates were similar at 12 and 24 months, however, P-IFNα-2a was associated with a higher rate of grade 3/4 toxicity [58]. Both arms appeared to induce comparable effects on the spleen size, karyotypic abnormalities, histopathological parameters, incidence of thrombotic complications and disease evolution, making them equally capable of modifying the natural history of high-risk ET/PV.

Resistance or intolerance to HU occurs in 25 to 35% of ET patients who then become at a higher risk of overall mortality and leukemic transformation [59]. A prospective, open-label, phase II clinical trial for treatment with P-IFNα was conducted by the MPN-RC across several sites in North America and Europe and included 65 ET patients who were either resistant or intolerant to HU [60]. Treatment with P-IFNα showed an overall response in approximately two-thirds of patients with high-risk ET and PV. Moreover, the presence of *CALR* mutations was associated with higher complete response rates to P-IFNα-2a suggesting a stronger indication for it when the mutational status is known [60].

A known frontline therapy for PV that has been recently introduced and studied in ET is the very long-acting monopegylated interferon, ropeginterferon alfa-2b (R-INFα-2b). A prospective, open-label, multicenter phase 1/2 dose-escalation study (PEGINVERA) showed that R-INFα-2b induced high response rates at a low dose and with minimal toxicity in patients with PV [61]. The drug also showed non-inferiority to HU in PV when given every 2 weeks followed by once monthly as per the 3-year follow-up data [62]. The non-inferiority to HU in terms of hematological response and normal spleen size was not evident at 12 months; however, improved responses happened at 36 months [62]. It has been also reported that R-INFα-2b can induce significant partial and complete molecular response rates, as reflected by the reduction of the *JAK2* allelic burden and more recently also of *TET2* [63]. R-INFα-2b is a promising medication for ET patients and is currently being investigated as a potential second-line therapy in the setting of HU resistance or intolerance. Presumably, future trials will be conducted to study it as a promising frontline treatment for ET as compared to HU or best available therapy (BAT).

### 4.3. Ruxolitinib 

Besides, a long-term phase 2 study of ruxolitinib, a *JAK1*/*2* inhibitor, demonstrated that patients with ET who are refractory to or intolerant of HU can achieve clinically meaningful and durable reductions in PC and WBC, and improvements in ET-related symptoms with this treatment [64]. When compared with BAT in a randomized phase II trial in patients resistant or intolerant to HU (MAJIC-ET), no significant difference in the rate of complete or partial hematologic remission was found [65]. In addition, the incidence rate of thrombosis, hemorrhage, and leukemic transformation at 2 years was not significantly different. However, symptomatic relief with improved itching and weight loss was reported with ruxolitinib [65]. Hence, the use of this drug remains controversial. Of note, the phase IIb RUXBETA trial that compared ruxolitinib vs. BAT in patients with high-risk ET refractory after first-line treatment (NCT02962388) has been abandoned. Ongoing clinical trials assessing ruxolitinib in adults with ET include: (i) phase 3 Ruxo-BEAT trial that compares ruxolitinib vs. BAT in patients with high-risk PV or high-risk ET(NCT02577926) and (ii) phase 2 RESET-272 trial with ruxolitinib vs. anagrelide in subjects with ET who are resistant to or intolerant of HU (NCT03123588). Perhaps, future trials will aim to understand the potential benefit and efficacy of combination therapies with ruxolitinib as compared to monotherapy.

### 4.4. Therapeutic-Choice Considerations 

In summary, cytoreductive agents showed comparable capabilities of achieving the primary endpoints of complete or partial hematologic remission, molecular response (a new therapy achievement in the NGS era), tolerability, and prevention of thrombosis. Perhaps the main influence on the physician’s therapeutic decision-making remains the patient’s circumstances, life events, and history. For example, P-IFNα is quite safer than HU for young female patients wishing to conceive [66,67,68,69,70]. The long-term follow-up of patients treated with these therapies in the clinical practice has helped describe the main adverse effects related to their use. For instance, HU can cause oral ulcers, skin hyperpigmentation, rashes, and teratogenicity [71]; anagrelide has been associated with fluid retention, headaches, heart palpitations, and cardiomyopathy [72,73]; P-IFNα can cause depression, flu-like symptoms, headache, malaise, fevers, arthralgia, pruritus, injection-site reactions, gonadal toxicity, and thyroid dysfunction. Other cytoreductive agents like pipobroman, busulfan, and radioactive phosphorus have been historically used but are reported to accelerate leukemic transformation [74].

## 5. Antiplatelet Therapy 

Beyond cytoreduction, antiplatelet therapy such as low dose ASA, has been indicated for microvascular disturbances and is beneficial in reducing the risk for thrombosis [34,75], preferentially in symptomatic patients and those with *JAK2* mutations. It can also reduce arterial thrombosis in patients with concurrent cardiovascular risks. On the contrary, in *CALR*-mutated ET patients, ASA may increase bleeding risk without reducing thrombosis, and this population is less likely to require cytoreduction because of a lower vascular complication risk [76]. Although, the antithrombotic effect of low-dose ASA in ET has not been evaluated in a randomized controlled trial, a systematic review of randomized and observational studies showed that the risk/benefit ratio of antiplatelet therapy in adults with ET is highly uncertain [77]. In addition, it is also a matter of debate whether the once/daily *vs*. twice/daily dosing regimen of ASA should be adopted. Currently, the once/daily dosing is recommended in lower-risk patients while the twice/daily in higher-risk patients, according to the revised IPSET score [33]. This notwithstanding, a universal posology for a safe and effective prophylaxis of vascular events is still controversial. The preliminary results from an Italian multicenter, double-blind study that included 245 ET patients, showed that the currently recommended ASA regimen of 75–100 milligrams (mg) daily for cardiovascular prophylaxis appears to inadequately reduce platelet COX-1 activation. Conversely, platelet inhibition was markedly improved by shortening the dosing interval to 12 h while preserving COX-2-dependent vascular thromboresistance [78].

## 6. Vitamin K Antagonists (VKA)

Apart from the primary prophylaxis of thrombosis, patients with ET also pose a challenge related to the acute treatment of venous thromboembolism (VTE) and whether the secondary prophylaxis after a first VTE episode should be finite or indefinite [79]. Similar to non-ET patients, low molecular weight heparin followed by VKA is the standard treatment as it has shown to be effective in the prevention of VTE recurrence in a cohort of 494 patients with PV or ET [hazard ratio (HR) 0.32, 95% CI: 0.15–0.64)]. [80] This notwithstanding, the annual incidence of rethrombosis in ET is higher than that in non-ET patients (7.8% vs. 1.8%-3.5% at 1 year, respectively) [81,82]. Moreover, the rate can double after the discontinuation of therapy as shown in an international study on 206 patients with MPN (HR 2.21, 95% CI: 1.19–5.30) [81]. Of note, the highest recurrence rate has been reported in ET patients experiencing unusual site thrombosis (splanchnic or cerebral venous sinus, 8 and 8.8 per 100 patients-year, respectively) [83,84]. The occurrence of major bleeding in ET (1.8–2.4 per 100 patients-year) is comparable to non-ET patients under VKA treatment [79,85]. However, the VKA combination with antiplatelet agents (mainly used during the first 6 months after a first VTE episode in particular cases) increases bleeding risk when compared to VKA alone (2.8% vs. 0.9%, respectively) [80]. Nonetheless, there remain paucity of studies and lack of consensus criteria or universal approach for VTE treatment in ET and a recent survey reported a high level of heterogeneity in the used therapeutic modalities [86]. In the future, controlled trials to optimize the use of VKA in ET, and more in general in Philadelphia chromosome-negative MPN patients, as well as the evaluation of safety and effectiveness of the new oral anticoagulants, are warranted. 

## 7. Therapeutic Interventions in Non-High-Risk Patients: A Matter of Controversial Debate

Traditionally, the mainly included population in ET clinical trials were the high-risk patients. Recently, an open-label randomized trial was conducted to compare HU plus ASA vs. ASA alone in patients with ET, aged 40 to 59 years, and without high-risk factors or extreme thrombocytosis [87]. The study showed that adding HU to ASA in these patients did not reduce vascular events, myelofibrotic transformation, or leukemic transformation. It also concluded that young low-risk patients without other clinical indications for treatment (e.g., previous thrombosis or hemorrhage), and who have a PC < 1500 × 10^9^/L, should not receive cytoreductive therapy. An interesting finding was that among patients treated with HU and ASA, major hemorrhages (intracranial, gastrointestinal or postoperative hemorrhages) occurred in 2% (*n* = 4) and minor hemorrhages in 15% (*n* = 26) of cases. However, their incidence rates were not significantly different from that of patients randomized to ASA alone (3%, (*n* = 5) and 14% (*n* = 25), respectively) [87]. In this context, a debate arises regarding the benefit of cytoreduction in patients without high-risk disease or prior history of bleeding but with extreme thrombocytosis (PC > 1000 × 10^9^/L). The dilemma is whether there is a beneficial effect of cytoreductive therapies on limiting the presumed risk of AVWS-related hemorrhage, as compared to observation. Unfortunately, there is no evidence-based practice data that could support either approach. Besides, in life-threatening situations with extreme thrombocytosis, a therapeutic intervention to consider for emergent reduction of PC is thrombocytapheresis. Although no current guidelines are set to draw stringent indications for such intervention, a case series reported on 4 ET patients who were successfully treated with thrombocytapheresis [88]. Thrombocytapheresis showed positive outcomes in scenarios of neurological symptoms due to microcirculatory disturbances, transient thromboembolic episode from hyperthrombocytosis, hyperthrombocytosis-related AVWS, prophylactic reduction of PC, and in symptomatic relief in the context of ischemia and simultaneous bleeding diathesis [88].

## 8. Extreme Thrombocytosis and Acquired von Willebrand Syndrome: The Paradox of Hemorrhagic Thrombocythemia

Since the first report in 1934, it has been recognized that extreme thrombocytosis (PC > 1000 × 10^9^/L) in ET may be associated with bleeding complications [89]. However, the paradox of a bleeding diathesis in a patient with high PC is a clinical conundrum and the correct identification and management of AVWS is still a matter of an individual clinical acumen among physicians.

AVWS is a rare bleeding disorder that is similar to its inherited counterpart in terms of clinical and laboratory manifestations. VWF is a large multimeric glycoprotein that is mostly produced and stored in Wiebel-Palade bodies in endothelial cells. While inactive in circulation, VWF is bound to factor VIII, which in turn protects it from bloodstream degradation. The facilitated interaction between platelets factor receptor GPIb and VWF due to high platelet numbers, as seen in ET, permits the degradation of VWF via ADAMTS13. More in general, AVWS is the result of low levels of VWF due to its accelerated removal from plasma by: (i) antibodies, as in the case of lymphoproliferative or immunologic conditions; (ii) in vivo absorption onto malignant cells, as in ET or solid tumors; (iii) conditions of high shear stress, as in cardiovascular disorders [90]. When the last two mechanisms are at play, a preferential removal of high molecular weight (HMW) VWF multimers occurs and results in a phenotype resembling type 2A or 2B VWF disease [91]. Thus, AVWS in ET is more characterized by a qualitative deficiency rather than a quantitative defect of VWF. This translates into normal levels of VWF:Ag (VWF antigen) with decreased ristocetin cofactor activity (VWF:RCoA usually below 20% in AVWS) and ultimately a VWF: RCoA/VWF:Ag ratio < 0.7 (normal value around 1) [92]. The pattern of VWF multimers is almost identical to type 2 VWD as depicted by multimeric analysis of patients with ET and extreme thrombocytosis before treatment with desmopressin (DDAVP) which corrects the VWF multimeric distribution [91] Therefore, patients with AVWS are prone to experience bleeding complications. 

**Figure 1:**
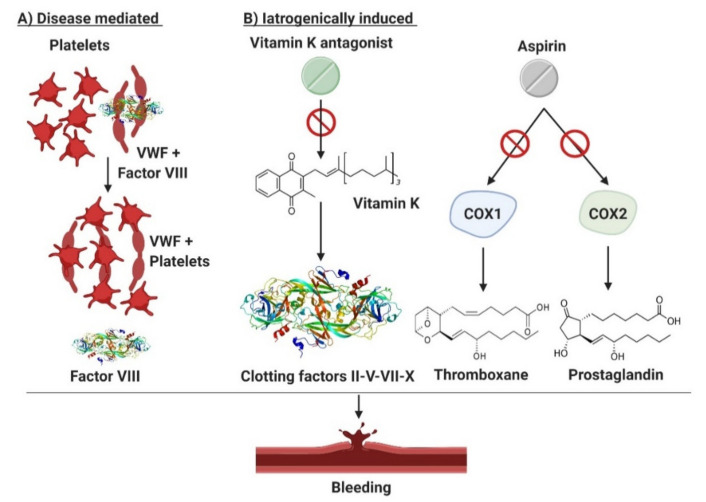
Bleeding mechanisms in patients with essential thrombocythemia. Patients with ET are prone to bleeding from AVWS or due to the use of antiplatelet/anticoagulant drugs. (**A**) illustrates the disease mediated mechanism in setting of excessive thrombocytosis which may cause AVWS due to in vivo absorption of VWF multimers. (**B**) describes iatrogenically induced bleeding risk in ET. Aspirin suppresses the production of thromboxane and prostaglandins by inactivating cyclooxygenase enzymes 1 and 2 (COX1/2) and thus impairing platelets aggregation. Vitamin K antagonist (used in selected patients with ET with active or prior thrombosis) have an intrinsic bleeding risk because of their narrow therapeutic window. Finally, excessive thrombocytosis may cause AVWS due to in vivo absorption of VWF multimers. COX1/2, cyclooxygenase enzymes type 1 and 2; VWF, von Willebrand factor.

The scenario is even more complicated when considering patients with ET presenting with extreme thrombocytosis who are often under ASA or anticoagulant treatment (Figure 1). This subset of patients shows a significantly prolonged Ivy bleeding time when compared to normal control and patients with reactive thrombocytosis (RT) [93]. The difference in bleeding time in RT and normal controls is explained by the thrombocytopathy of patients with ET. Malignant platelets are hypersensitive, activated by the high shear stress in the microcirculation, and show a secondary storage pool disease with pseudopodia and centralized granules [75]. Based on these insights, the use of antiplatelet agents, a common clinical practice in patients with ET as aforementioned, requires caution. Indeed, situations of extreme thrombocytosis may render clinicians’ choices a cause of anxiety due to an unpredictable thrombo-hemorrhagic balance. 

## 9. Incidence Rates and Risk Factors of Bleeding in Essential Thrombocythemia with Extreme Thrombocytosis 

While thrombosis is very well discussed in MPN, only few studies reported on the incidence rates and risk factors of bleeding (Table 3). Historically, extreme thrombocytosis and subsequent AVWS have correlated with the paradoxical hemorrhagic complication in patients with ET. The International Registry on AVWS data shows that lymphoproliferative and myeloproliferative disorders account for almost 50% of cases of AVWS [90]. In addition, more than 50% of patients with MPN, including ET and PV, display laboratory evidence of AVWS that sometimes becomes clinically evident with mucocutaneous bleeding diathesis [94].

One of the largest series published on AVWS revealed interesting insights on the peculiarities of patients with ET who develop this complication [95]. MPN, as an underlying disorder, was three times more common among patients who had bleeding complications compared with lymphoproliferative diseases; however, the bleeding tendency was more common in non-MPN patients who in turn also experienced thrombotic complications. Thus, ET displays a particular scenario where bleeding and thrombosis are simultaneous manifestations of the hemostatic disequilibrium. 

In another series of 99 low-risk young patients with ET and extreme thrombocytosis (defined as PC > 1000 × 10^9^/L), Tefferi et al. did not find any difference in the prevalence of thrombotic nor hemorrhagic complications. The results were also not different when excluding patients with or without cytoreductive therapy, using a cut-off age of 40 years nor when excluding those with microvascular symptoms from the overall analysis [96].

An Italian retrospective analysis of 565 patients with ET reported a 14% cumulative risk of hemorrhage at 10 years [97]. The median PC and WBC at the time of bleeding was 560 × 10^9^/L (range, 122–1322 × 10^9^/L) and 7.3 x10^9^/L (range, 4.8–12.7 × 10^9^/L), respectively and the majority of patients (77%) were on antiplatelet and/or anticoagulant agents. The multivariate analysis showed prognostic significance for PC > 1000 × 10^9^/L, WBC > 11 × 10^9^/L, and splenomegaly in terms of bleeding complication. These results are very important especially when considering that the most common site of bleeding was the gastrointestinal tract (in about 50% of the cases). Patients with splenomegaly may develop portal hypertension, which can contribute to an additive bleeding risk factor for an already unbalanced thrombo-hemorrhagic homeostasis. Moreover, this study emphasized the role of leukocytosis in patients experiencing bleeding complications. A U-shaped relationship between WBC and risk of hemorrhage was also found in the PT-1 study cohort, with the hazard of bleeding increasing simultaneously with WBC [28].

Another international study reviewed 1104 patients according to WHO 2008 histological criteria and confirmed ET diagnosis in 891 patients while 180 patients were classified as PFMF [98]. When focusing on the bleeding incidence and risk factors, a multivariable analysis found that the diagnosis of PFMF, leukocytosis, previous hemorrhage, and ASA use were predictors of such complication. The analysis, which was restricted to ET WHO-confirmed patients, found that previous hemorrhage and ASA use were independent risk factors. Patients in the spectrum of ET/PFMF had higher WBC (>11 × 10^9^/L) and PC (>1000 × 10^9^/L) at diagnosis as well as major bleeding tendency during follow-up (12% vs. 6% in ET-WHO confirmed cases, *p* = 0.009). Of note, in both groups, similarly to Palandri et al [97], the most common bleeding site was the gastrointestinal tract. Here, we may argue that these findings, together with the report of splenomegaly as a bleeding risk factor in the preceding study, may be the result of clinically unrecognized portal hypertension that may cause a *situs minoris resistentiae* in the gastrointestinal tract. The higher pressure in the splanchnic vascular system and the dysfunctions in terms of the quality and number of platelets may generate an unbalanced rheologic and hemostatic status resulting in bleeding complications. 

Moreover, while the role of PC in affecting VWF multimers and in the pathophysiology of AVWS has been described, it is still not fully explained how leukocytes contribute to the occurrence of bleeding. MPNs are characterized by increased WBC and multiple studies have shown their role as a risk factor for these fatal complications. Activated neutrophils have been associated with blood-brain barrier impairment and extracellular matrix destruction resulting from metalloproteases and elastase release [99,100]. Nevertheless, Finazzi et al. showed that the major bleeding risk factor in a population of WHO-defined ET was ASA use [98]. This statement goes in line with previous reports which demonstrated that the use of antiplatelet agents in patients with low-risk ET and extreme thrombocytosis was associated with increased risk of bleeding [101].

Finally, it is striking how AWVS has been also related to near-normal PC as described by Tefferi et al. [92]. In their case report, the authors discussed a case of a 61-year-old patient with PV who had mucocutaneous bleeding diathesis despite a PC of 488 × 10^9^/L. In this scenario, although the role of PC cannot be completely excluded, the high shear stress from increased blood viscosity may have played a major pathogenetic contribution. That said, the treatment of the underlying disorder eventually led to the resolution of the AVWS. 

## 10. Options for Therapeutic Management of Acquired von Willebrand Syndrome in Essential Thrombocythemia

Whilst the management of AVWS is more evident in cases of lymphoproliferative, cardiologic, and immunologic disorders, less is known about it when associated with ET with extreme thrombocytosis where the bleeding tendency is counteracted by the intrinsic risk of thrombosis. As previously discussed, there exists evidence that supports the role of platelets in AVWS as the decrease in their count can lead to the normalization of laboratory abnormalities [102,103]. Hence, cytoreduction strategies and thrombocytapheresis have been routinely used in emergency situations [104]. Although no treatment is indicated for subclinical cases of AVWS, however, in cases with bleeding complications or surgical requirements, AVWS may be treated as its inherited counterpart with DDAVP and or FVIII/VWF concentrates [105]. Immunosuppressive drugs, immunoglobulin infusions, and plasma exchange are more considerable options for AVWS in lymphoproliferative disorders where the pathogenetic mechanism at play is the presence of an acquired antibody.

**Figure 2:**
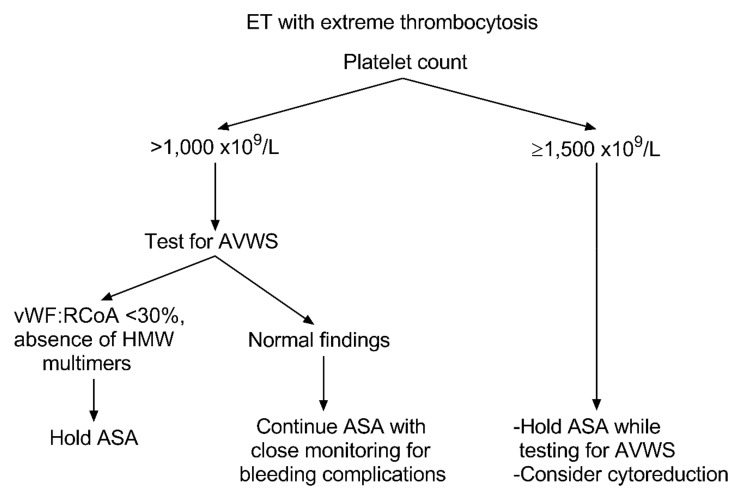
Approach to aspirin use in essential thrombocythemia with extreme thrombocytosis. The algorithm explains how to approach extreme thrombocytosis (platelet count > 1000–1500 × 10^9^/L) in essential thrombocythemia. It illustrates the investigational steps required for testing acquired von Willebrand syndrome in accordance with aspirin use, in a timely manner. ET, essential thrombocythemia; AVWS, acquired von Willebrand syndrome; HMW, high molecular weight; VWF:RCoA, von Willebrand factor ristocetin cofactor activity; ASA, aspirin.

Notwithstanding that cytoreduction is not a matter of debate in high-risk patients with extreme thrombocytosis, concerns might arise regarding the use of antiplatelet agents in young patients with low-risk ET and PC > 1000 × 10^9^/L. Here, a peculiar situation may be useful to emphasize this point: pediatric ET. Many reports described that children with ET present with massive thrombocytosis (PC ranging up to 3–4000 × 10^9^/L or more) and are prone to bleeding complications due to platelet dysfunction [106,107]. As for the case of acute lymphoblastic leukemia treatment, adult hematologists may learn from pediatricians on how to manage these cases of low-risk young patients with ET and extreme PC. The aphorism “primum non nocere” explains the concerns related to the use of antiplatelet agents as it can likely increase the risk of hemorrhagic complications. Indeed, bleeding occurs less commonly in patients younger than 40 years. In this scenario, the patients’ PC and their individual thrombo-hemorrhagic risk factors are crucial for guiding clinicians’ consideration to test for AVWS. For instance, The British Committee for Standards in Hematology considers a PC threshold of 1500 × 10^9^/L as an indication for cytoreduction and recommends cautious use of ASA in patients with a history of bleeding or extreme thrombocytosis (PC > 1000–1500 × 10^9^/L) [67]. Similarly, the German and Austrian Haematology and Oncology Societies emphasize to perform a functional analysis of VWF:Ag, VWF:RCoA, and others in patients with PC > 1000 × 10^9^/L. They also advise avoiding antiplatelet therapy in patients with abnormal findings suggestive of AVWS (VWF:RCoA ≤ 30%) (Figure 2) [108]. However, as previously noted, the most crucial laboratory finding for diagnosing AVWS in ET is the VWF multimers’ pattern analysis. Despite its importance, a limitation of its wide use is the need for a laboratory with high specialization in coagulation.

## 11. Conclusions

In conclusion, it seems reasonable to adopt the approach of lowering PC and administrating antiplatelet agents in high-risk patients with ET to counteract the intrinsic thrombotic risk. In low-risk patients, PC > 1000 × 10^9^/L entitles closer evaluation in order to identify subclinical AVWS cases. Finally, low-risk patients with PC ≥ 1500 × 10^9^/L should avoid antiplatelet agents and may benefit from cytoreductive treatment in a personalized fashion. 

## Figures and Tables

**Table 1:** The evolution of essential thrombocythemia diagnostic criteria according to World Health Organization (WHO).

WHO 2008	WHO 2016 (Revised)
**Major criteria**	**Major criteria**
1. Platelet count ≥ 450 × 10^9^/L	1. Platelet count ≥ 450 × 10^9^/L
2. BM biopsy showing proliferation of the megakaryocyte lineage with increased numbers of enlarged, mature megakaryocytes	2. BM biopsy showing proliferation of the megakaryocyte lineage with increased numbers of enlarged, mature megakaryocytes with hyperlobulated nuclei. No significant left-shift of neutrophil myelopoiesis or erythropoiesis and very rarely minor (grade 1) increase in reticulin fibers
3. Not meeting WHO criteria for CML, PV, PMF, MDS, or other myeloid neoplasms	3. Not meeting WHO criteria for BCR-ABL1 + CML, PV, PMF, MDS, or other myeloid neoplasms
4. Presence of *JAK2* V617F mutation or other clonal marker or lack of evidence of a secondary cause of thrombocytosis	4. Presence of *JAK2*, *CALR* or *MPL* mutation
No minor criteria	**Minor criteria** 1. Presence of a clonal marker or absence of evidence for reactive thrombocytosis
All four major criteria required	All four major criteria or three major and one minor required

Adapted from Arber et al. [2]. Abbreviations: BM, bone marrow; CML, chronic myeloid leukemia; PV, polycythemia vera, PMF, primary myelofibrosis; MDS, myelodysplastic syndromes.

**Table 2:** Evolution of prognostication systems in essential thrombocythemia.

**Traditional ELN Guidelines**
(a) High-risk: age ≥ 60 years or previous thrombosis
(b) Low-risk: none of the above
**IPSET-thrombosis**
Risk factors: Age > 60, = 1 point; Cardiovascular risk factors (tobacco use, diabetes, hypercholesterolaemia, hypertension), = 1 point; Previous thrombosis, = 2 point; *JAK2* V617F, = 2 points
(a) Low risk: 0–1
(b) Intermediate risk: 2
(c) High-risk: ≥3
**IPSET- thrombosis (Revised)**
(a) Very low risk: no thrombosis history, age ≤ 60 years and *JAK2*/*MPL*-unmutated
(b) Low risk: no thrombosis history, age ≤ 60 years and *JAK2*/*MPL*-mutated
(c) Intermediate risk: no thrombosis history, age > 60 years and *JAK2*/*MPL*-unmutated
(d) High risk: thrombosis history or age > 60 years with *JAK2*/*MPL* mutation
**MIPSS-ET**
Risk factors: Adverse mutations (*SRSF2*, *SF3B1*, *U2AF1* and *TP53*) = 2 points; age > 60 years = 4 points, male sex = 1 point and leukocyte count ≥ 11 × 10^9^/L = 1 point
(a) Low risk: 0–1
(b) Intermediate risk: 2–3
(c) High-risk: ≥4

**Table 3:** Risk factors for bleeding in patients with essential thrombocythemia.

Risk Factors
Advanced age (>60 years)
Extreme thrombocytosis (platelet count > 1000 × 10^9^/L)
Leukocytosis (leukocyte count ≥ 11 × 10^9^/L)
Driving mutation: *JAK2* V617F is associated with higher risk of bleeding (*CALR* unclear)
History of bleeding/thrombosis
Acquired von Willebrand syndrome
Splenomegaly and portal hypertension
Medication induced (antiplatelet therapy and anticoagulation therapies)
